# Enhanced efficacy of sitravatinib in metastatic models of antiangiogenic therapy resistance

**DOI:** 10.1371/journal.pone.0220101

**Published:** 2019-08-01

**Authors:** Melissa Dolan, Michalis Mastri, Amanda Tracz, James G. Christensen, Gurkamal Chatta, John M. L. Ebos

**Affiliations:** 1 Department of Experimental Therapeutics, Roswell Park Comprehensive Cancer Center, Buffalo, New York, United States of America; 2 Department of Cancer Genetics and Genomics, Roswell Park Comprehensive Cancer Center, Buffalo, New York, United States of America; 3 Department of Medicine, Roswell Park Comprehensive Cancer Center, Buffalo, New York, United States of America; 4 Mirati Therapeutics, San Diego, California, United States of America; European Institute of Oncology, ITALY

## Abstract

Tyrosine kinase inhibitors (TKIs) that primarily target angiogenesis are approved to treat several cancers in the metastatic setting; however, resistance is common. Sequential treatment or ‘switching’ from one TKI to another following failure can be effective, but predicting which drugs will have cross-over sensitivity remains a challenge. Here we examined sitravatinib (MGCD516), a spectrum-selective TKI able to block MET, TAM (TYRO3, AXL, MerTK) and multiple receptor families (including PDGFRs, VEGFRs, and Ephs). Transcriptomic analysis of several mouse and human cell lines revealed diverse molecular changes after resistance to two TKIs (sunitinib and axitinib) with multiple sitravatinib targets found to be upregulated. Sitravatinib treatment *in vitro* resulted in enhanced anti-proliferative effects in resistant cells and was improved compared to TKIs with similar target profiles. *In vivo*, primary tumor growth inhibition after sitravatinib treatment in mice was enhanced in resistant tumors and metastasis suppression improved when tumors were surgically removed. Together, these results suggest that the diverse and often inconsistent compensatory signaling mechanisms found to contribute to TKI resistance may paradoxically improve the tumor-inhibiting effects of broad-spectrum TKIs such as sitravatinib that are able to block multiple signaling pathways. Sitravatinib in the second-line setting following antiangiogenic TKI treatment may have enhanced inhibitory effects in local and disseminated disease, and improve outcomes in patients with refractory disease.

## Introduction

Multiple small molecule receptor tyrosine kinase inhibitors (TKIs) targeting tumor angiogenesis are approved for first- or second-line treatment in several metastatic cancers [1]. But such TKIs are rarely specific and broad ‘off-target’ treatment effects are common [2]. This may explain the diverse patient toxicities observed for TKIs in similar drug classes [3–5] and why resistance to one TKI does not necessarily diminish the efficacy of another [6]. Indeed, alternating or ‘switching’ TKI treatments occurs in several clinical settings [6]. For example, TKIs primarily targeting vascular endothelial growth factor receptors (VEGFRs) such as pazopanib, sorafenib, axitinib, and lenvatinib (with an mTOR inhibitor), are approved as second-line treatments following failure to sunitinib or sorafenib [7]. Such instances of cross-over sensitivity may be due to variations in drug-specific target profiles [6]. For example, most VEGFR TKIs also target PDGFRs, and others have sensitivities to FGFRs (nintedanib, regorafenib), TIE receptors (regorafenib), c-KIT (dovitinib, pazopanib), RET (sorafenib), CSF-1R (linifanib), among others [8]. In this regard, TKIs targeting MET and AXL (i.e., cabozantinib) have been particularly effective in preclinical resistant settings [9–13] and found to improve progression-free and overall survival in patients after sunitinib and sorafenib treatment failure. However, predicting which TKI may have benefits after resistance remains a challenge. TKI-resistance can include compensatory signaling involving MET, AXL, FGF, (amongst others) [14, 15]; but preclinical testing rarely considers secondary TKI treatments in models with broad (often inconsistent) resistance-cell profiles that more accurately resemble the diversity observed in the clinical setting [1, 6, 16].

In this study, we examined the activity of sitravatinib (MGCD516), a multi-spectrum TKI in preclinical resistance models derived from complex tumor stages (i.e., primary tumors and metastasis after surgery), multiple cell-origin types (i.e., breast and kidney carcinomas), different species (human and mouse), and varied inhibitors (sunitinib and axitinib). Sitravatinib has recently been shown to inhibit MET in mesothelioma and lung cancers, PDGFRs in sarcomas [17, 18], and select TAM (**T**YRO3, **A**XL, **M**erTK) receptors in tumor associated macrophages, amongst several other receptor families [19, 20]. Currently, sitravatinib is being evaluated in Phase 1/2 clinical trials involving multiple cancers after prior treatments, including VEGFR TKIs (NCT03015740). Uniquely, sitravatinib also has inhibitory activity against erythropoietin-producing hepatocellular carcinoma (Eph) family members. This includes receptors (EphA and EphB) and ligands [17] that are expressed on tumor and stromal cells [21] and are capable of mediating VEGF signaling and metastasis [22–26].

Due to this broad activity, we examined sitravatinib treatment effects in several highly variable models of TKI resistance that include clinically-relevant metastasis, something rarely performed in mice (see [27] for review). This included resistant cell lines derived from spontaneous metastasis occurring after the surgical resection of orthotopically implanted tumors [28]. Using transcriptomic and proteomic analysis, TKI resistant cells were found to have multiple sitravatinib targets upregulated, including MET, AXL, and Eph family members, but these were highly diverse amongst cell lines tested. These complex resistance phenotypes may explain the enhanced anti-proliferative effects of sitravatinib observed *in vitro* when compared to non-resistant control cells and to cabozantinib, which has a similar target profile. Critically, sitravatinib treatment showed enhanced inhibitory effects of primary tumor growth and metastasis (after primary tumor removal) in resistance models *in vivo*, suggesting prior TKI treatment may boost drug potency. Together, these studies provide a rationale for sitravatinib as a second-line treatment after antiangiogenic TKI therapy failure.

## Materials and methods

### Cell lines

Human breast carcinoma LM2-4 cells (a metastatic derivative of MDA-MB-231) and mouse kidney carcinoma RENCA cells express luciferase (alternatively termed LM2-4^LUC+^ and RENCA^LUC+^), and have been described previously [29–31]. Mouse mammary carcinoma 4T1 cells and mouse fibroblast 3T3 cells were gifts from A. Gudkov and I. Gelman, respectively. Cells were maintained in DMEM (Corning cellgro #10-013-CV for LM2-4 and 3T3) or RPMI (Corning cellgro #10-040-CV for 4T1 and RENCA). Growth media were supplemented with 5% v/v FBS (Corning cellgro; 35-010-CV). Cells were maintained at 37°C with 5% CO_2_ in a humidified incubator. Human cells were authenticated by STR profile comparison to ATCC cell database and mouse cells were confirmed of species origin (DDC Medical, USA).

### Drugs and concentrations used

SU11248/Sunitinib malate (Sutent, Pfizer), AG013736/Axitinib (Inlyta, Pfizer), Sitravatinib (Sitravatinib, Mirati Therapeutics), and Cabozantinib (LC Labs; C-8901) were used as described previously by us [2, 30] and others [10, 17]. For *in vivo* treatments, sitravatinib was suspended in vehicle formulation containing PEG300 (40% v/v) and 0.1N HCl in normal saline (60% v/v). In some experiments, vehicle groups included mice treated with sitravatinib-vehicle or sunitinib-vehicle as controls (sunitinib-vehicle formulations were described previously [2]). No tumor-related differences between any vehicles were observed. Mice received 20 mg/kg/day sitravatinib by oral gavage as recommended by the manufacturer [2]. For *in vitro* maintenance of resistant cell lines, sunitinib was dissolved in water (1mM stock solutions) and axitinib was dissolved in DMSO (10mM stock solutions). For experiments, sitravatinib and cabozantinib were dissolved in DMSO (10mM stock solutions) as recommended by the manufacturers. All *in vitro* experiments using sitravatinib and cabozantinib included DMSO and are referred to as ‘control’ or ‘vehicle-treated’ in this study.

### Ortho-surgical mouse models of metastasis

Animal studies were performed in strict accordance with the recommendations in the Guide for Care and Use of Laboratory Animals of the National Institutes of Health and according to guidelines of the Institutional Animal Care and Use Committee (IACUC) at Roswell Park Comprehensive Cancer Center (RPCCC). All studies were approved by the IACUC at RPCCC according to Protocol 1227M. All personnel involved in this study were included in the IACUC protocol and approved/trained by veterinary staff to conduct all experimental procedures described.

#### Ortho-surgical models of metastasis

LM2-4 (1x10^6^ cells in 100μl DMEM), 4T1 (4x10^4^ cells in 100μl RPMI), or RENCA (5x10^4^ cells in 2.5μl RPMI and 2.5μl matrigel) were implanted orthotopically into the right inguinal mammary fat pad (right flank) or left kidney (subcapsular space) of 6–8 week old female SCID or Balb/c mice depending on the model [30]. Primary breast tumor volume was assessed with Vernier calipers using the formula (width^2^×length)×0.5 and, for tumor cells expressing luciferase, animals were monitored bi-weekly for bioluminescence (BL) [32]. Surgical removal of breast tumors, as well as nephrectomy of tumor-bearing kidneys, was performed using procedures optimized by us previously [30, 31, 33]. This included the selection of surgical time points aimed to minimize primary tumor invasion to adjacent organs while maximizing metastatic disease distribution [30]. All surgeries were performed under anesthesia (isoflurane), and analgesic (buprenorphine) was administered during recovery as per approved IACUC protocol guidelines. Animals were monitored 2–3 times daily by veterinary staff and IACUC-approved personnel, with increased daily monitoring (4 times) if animals presented with ruffled fur, weight loss, ocular discharge, lethargy, hunched back, inappetence, ataxia, tremors, ulcerated or infected tumors, diarrhea, huddled appearance, respiratory rate change, jaundice, and/or limb use impairment. Animals were sacrificed by cervical dislocation followed by necropsy within 24 hours when end-stage metastatic disease was reached. End-stage metastatic disease was defined in approved RPCCC IACUC protocols and in prior published protocols by us (see [31]). Endpoints included signs of distress, labored breathing, 20% weight loss, cachexia, lack of response to noxious stimuli, limb paralysis, or if present, measurable metastatic tumor growth at or near institutional size limits [30, 31, 33]. Animals with no signs of end-stage metastatic disease were euthanized at the end of the experiment and necropsy conducted.

#### Inclusion/Exclusion criteria

During surgery, if primary tumor invaded the adjacent tissues—i.e., growth into peritoneal space (breast) or a non-encapsulated tumor was found (kidney)–the mouse was excluded from study if complete removal of all visible tumor was not possible [33]. Additionally, if a vehicle-treated tumor was not present at any time before and after surgery (determined by BLI or visible macroscopically), mice were excluded from study to eliminate the potential of false-positive or false-negative results [30].

#### Randomization

Mice were randomized either i) before implantation (in studies where treatment started immediately after implantation) or ii) after tumor growth was measurable by primary tumor size to ensure equal tumor burden and prior to treatment (described in [30]).

### Derivation and maintenance of TKI-resistant cells

The generation of sunitinib and axitinib-resistant (SuR/AxR) cells have been described previously [28]. Briefly, human LM2-4 and mouse RENCA or 4T1 cells were implanted orthotopically into SCID or Balb/c mice. Mice were treated with sunitinib (60 mg/kg/day) or axitinib (100 mg/kg/day) during primary tumor growth and, following surgical removal of primary tumor or tumor-bearing kidney, treatment was continued until endpoint. At endpoint, spontaneous metastatic lesions were excised (from lung or lymph node), were disassociated, and then adapted to cell culture with continued 5μM sunitinib or 0.5μM axitinib treatment until experimental use. The inclusion of resistant tumor cell variants derived from lung metastasis is important because of recent studies showing that one mechanism of resistance to antiangiogenic therapies include vessel co-option by tumor cells in the lungs whereby the lack of nascent angiogenesis renders treatment to be less effective [34]. For *in vivo* studies, resistant cells were washed with PBS and then implanted. For *in vitro* studies, resistant cells were serum starved for 3 hours prior to sitravatinib treatment. 3T3^SuR^ variants were selected *in vitro* through incremental weekly increases of sunitinib up to 5μM, as previously described [28]. Sitravatinib, cabozantinib, sunitinib, or axitinib were not combined in any studies.

### Cell proliferation assay

Cell proliferation was measured using the CellTiter 96 Aqueous Non-Radioactive cell proliferation (MTS) assay (Promega; G1112) as described previously [28]. Briefly, 5x10^3^ and 1x10^3^ cells/well (for human and mouse SuR/AxR cell lines, respectively) were plated in 96-well plates in growth media. The next day, growth media was removed and replaced with media containing various concentrations of sitravatinib or cabozantinib. Sitravatinib concentrations ranged between 1 and 10μM for human and 0.02 and 10μM for mouse cell lines, and cabozantinib concentrations ranged between 0.01 and 10μM for mouse cell lines. Preliminary studies were conducted using multiple concentration ranges to arrive at optimal concentrations for IC50 determination. After 72 hours, MTS was added to the cells and, after 2 hours, measured at a wavelength of 490nm using a spectrophotometer (Bio-Rad xMark). Data were normalized to vehicle-treated controls and proliferation curves created to calculate the IC50 for each cell line.

### Annexin V staining

Cell apoptosis was quantified by Annexin V staining according to manufacturer’s protocol (Biolegend, 640930) [35]. Cells were plated in 6-well plates in their corresponding growth media (3 x 10^5^ cells/well). The next day, growth media was removed and replaced with 2μM sitravatinib or DMSO (for vehicle-treated controls). The choice of 2μM sitravatinib was based on IC50 data and used to maximize the observation of potential differences between P and SuR/AxR cell variants. After 48 hours, cells were washed, trypsinized, and stained with Annexin V. 7AAD cell viability stain (Biolegend, 420403) was used eliminate necrotic cells from the analysis. Positive controls for Annexin V included 1μM staurosporin (18 hour treatment) and 7AAD included incubation in a hot water bath (45 min; 55°C). Cells were analyzed using a LSRII flow cytometer (Becton Dickinson) and data acquired with FACSDiva software (Becton Dickinson). Data were analyzed with FCS Express 6 (De Novo Software).

### Western blot analysis

Samples were harvested with lysis buffer (50mM Tris (pH8), 2% w/v SDS, 5mM EDTA, 3mM EGTA, 25mM NaF, 1mM Na_3_VO_4_) supplemented with 1mM PMSF, 10μg/ml aprotinin, and 10μg/ml leupeptin or Halt^™^ Protease Inhibitor Single-Use Cocktail (Thermo Scientific; 78430). Following sonication (2 seconds) and centrifugation, cell supernatants were collected and total protein concentration was measured with DC protein assay (Bio-Rad, 5000112). Protein samples were diluted to equal concentrations and mixed with SDS-PAGE sample buffer (50mM Tris pH6.8, 2% w/v SDS, 5% v/v glycerol, 100mM DTT, and bromophenol blue). Proteins (30–45 μg/lane) were resolved by SDS-PAGE, electrotransferred to Immobilon-P membrane, and incubated with a primary antibody diluted as recommended by the manufacturer. Membranes were then probed with a horseradish peroxidase-conjugated secondary antibody (Promega, W4011, W4021, and V8051) and protein signals were developed using the Pierce ECL Western blotting substrate (Thermo Scientific; 32106) or the SuperSignal West Femto Maximum Sensitive Substrate (Thermo Scientific; 34095). X-ray films were imaged (digitized) with ChemiDoc System and analyzed with Image Lab Software (Bio-Rad). Primary antibodies were purchased from Cell Signaling (phospho-MET, 3126; phospho-EphA2, 12677; phospho-EphA3, 8862; MET, 3127; EphA2, 6997; EphA3, 8793), R&D systems (phospho-AXL, AF2228-SP; AXL, AF854-SP), and Sigma-Aldrich (α-tubulin, T6074). Uncropped original western blots are shown in S1 Appendix.

### RNA isolation

Cells were placed in media (1 x 10^6^ cells per 100mm plate) and RNA isolated after 48 hours using a QIAshredder (QIAGEN; 79654) and RNase mini kit (QIAGEN; 74104). Genomic DNA was eliminated by DNase I (QIAGEN, 79254) treatment as described in the on-column DNase digestion protocol from QIAGEN. RNA concentration was determined using nanodrop 2000c (Thermo Scientific).

### Whole genome expression analysis

Whole transcriptome profiling for RENCA, 3T3, and LM2-4 cell variants used Illumina mouse and human expression BeadChips to analyze P and SuR cell variants as has been described by us previously and deposited to the Gene Expression Omnibus (GEO) with accession numbers GSE122821, GSE122820, and GSE122819, respectively [28]. For 4T1 P/AxR cell variants used in this study, RNA sequencing analysis was performed using the Genomics shared resource at Roswell Park Comprehensive Cancer Center. Initially, the sequencing libraries were prepared with the TruSeq Stranded mRNA kit (Illumina Inc), from 1μg total RNA, following manufacturer’s instructions to select for PolyA RNA. After PolyA selection, the remaining RNA was purified, fragmented and primed for cDNA synthesis. Fragmented RNA was then reverse transcribed into first-strand cDNA using random primers. The RNA template was removed and a replacement strand was synthesized, incorporating dUTP in place of dTTP to generate ds cDNA. AMPure XP beads (Beckman Coulter) were used to separate the ds cDNA from the second-strand reaction mix resulting in blunt-ended cDNA. A single ‘A’ nucleotide was then added to the 3’ ends of the blunt fragments. Multiple indexing adapters, containing a single ‘T’ nucleotide on the 3’ end of the adapter, were ligated to the ends of the ds cDNA, preparing them for hybridization onto a flow cell. Adapter ligated libraries were amplified by PCR, purified using Ampure XP beads, and validated for appropriate size on a 4200 TapeStation D1000 Screentape (Agilent Technologies, Inc.). The DNA libraries were quantitated using KAPA Biosystems qPCR kit, and were pooled together in an equimolar fashion, following experimental design criteria. Each pool was denatured and diluted to 2.4pM with 1% PhiX control library added. The resulting pool was then loaded into the appropriate NextSeq Reagent cartridge, as determined by the number of sequencing cycles desired, and sequenced on a NextSeq500 following the manufacturer’s recommended protocol (Illumina Inc.). Sequencing quality control was assessed using FASTQC v0.11.5 (http://www.bioinformatics.babraham.ac.uk/projects/fastqc/). Reads were aligned to the mouse genome GRCm38 M16 (Gencode) using STAR v2.6.0a [36] and post-alignment quality control was assessed using RSeQC v2.6.5 [37]. Aligned reads were quantified at the gene level using RSEM v1.3.1 [38]. RSEM estimated gene counts were filtered and upper quartile normalized using the R-based Bioconductor package edgeR [39]. The 4T1 P/AxR raw and processed data were deposited in GEO (accession number: GSE132568).

### Statistical analysis

Statistical analysis was performed using the GraphPad Prism software package v.7.04 (GraphPad software Inc., San Diego, CA) and R v.3.4.1 through RStudio v.1.0.143 (Integrated Development for R; RStudio, Inc., Boston, MA). One sample t-test or Student’s two-tailed unpaired t-test was used for two-group comparison. For cell proliferation studies, IC50 was calculated using a four-parameter logistic curve and IC50 comparisons were determined by a sum-of-squares F test. Comparison of primary tumors (by caliper or BLI measurement) utilized a student’s two-tailed unpaired t-test. Kaplan-Meier methods were used to analyze overall survival with two-sided log-rank test to compare treatment. Descriptions for combined pre- and post-surgical summary analysis have been described previously [30]. Briefly, resected tumor (or tumor-bearing kidney) weights and overall survival were normalized to vehicle-treated animals to calculate percent difference. Then, student’s two-tailed unpaired t-tests were performed to separately compare primary tumor burden and overall survival of parental- and resistant-cell variants. A minimum significance level of 0.05 was used for all analyses.

## Results

### Antiangiogenic TKI resistance increases expression and activation of sitravatinib targets

**Fig 1:**
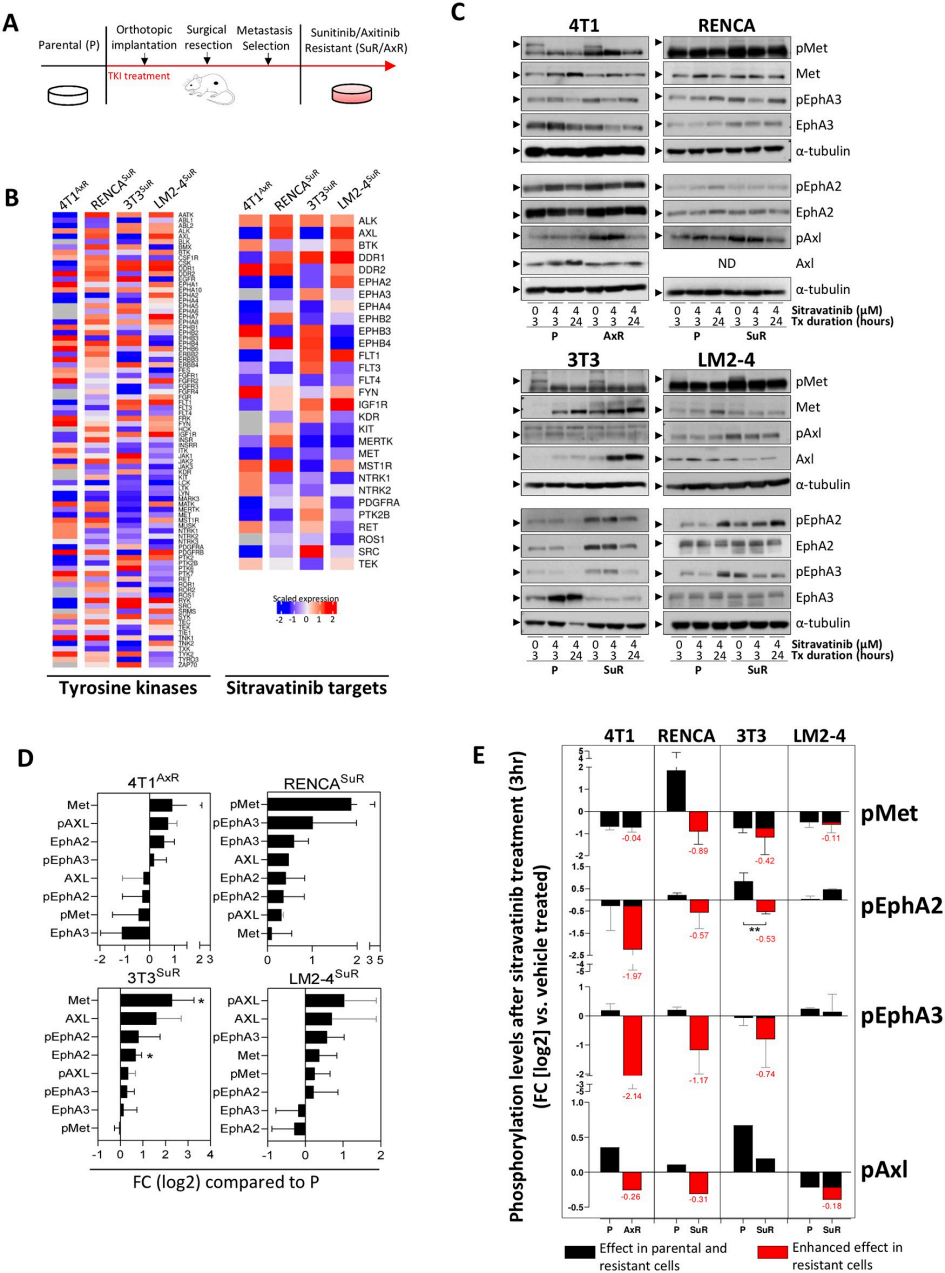
Sitravatinib target expression and activation increases in TKI-resistant metastatic tumor and non-tumor cells. (A) Schematic showing *in vivo* derivation of SuR and AxR cell lines from metastatic lesions following surgical removal of primary tumors. (B) Whole genome expression analysis showing tyrosine kinase and sitravatinib target genes in 4T1^AxR^, RENCA^SuR^, 3T3^SuR^, and LM2-4^SuR^ cells compared to corresponding P cells (n = 3). (C) Representative western blots of phosphorylated and total protein levels of Met, EphA2, EphA3, and Axl in 4T1, RENCA, 3T3, and LM2-4 cells for P or SuR/AxR variants after treatment with 4μM sitravatinib for 3 or 24 hours. (D,E) Densitometric analysis of protein levels. Both phosphorylated and total protein levels were normalized to α-tubulin. (D) Protein levels in SuR/AxR variants were compared to corresponding P cells (n = 1–5). (E) Phosphorylation levels after treatment with 4μM sitravatinib for 3 hours (n = 1–3) compared to vehicle (DMSO) treated cells. Red area in bar graphs represent instances where sitravatinib treatment effect on phosphorylation levels is enhanced in SuR or AxR cells compared to sitravatinib treatment effect in P cells. Red numbers represent the enhanced effect (FC; log_2_) in SuR or AxR cells compared to P cells after sitravatinib treatment. *P*, *parental; SuR*, *sunitinib resistant; AxR*, *axitinib resistant; FC*, *fold-change; TKI*, *tyrosine kinase inhibitor; Tx*, *treatment*. *ND*, *not done; n = 1*, *No error bars shown*, *n = 2*, *gray error bars shown; n>2*, *black error bars shown*. *Mean ± standard deviation (SD)*. *3T3*^*SuR*^ *mouse fibroblast cells were derived in vitro by concentration escalation as described in ref 28*. *See* S1 Appendix *for original uncropped images of western blots shown*. * *p<0*.*05*, ** *p<0*.*01*, *compared to P cells*.

To evaluate sitravatinib following antiangiogenic treatment failure, we performed transcriptomic and proteomic analysis on tumor and non-tumor cell lines resistant to sunitinib (Su) and axitinib (Ax). Su- and Ax-resistant cell lines (SuR and AxR, respectively) were derived from metastatic lesions spontaneously arising after surgical resection of a primary tumor following continuous treatment (schematic shown in Fig 1A; described previously [28]). Cell lines included human (LM2-4) and mouse (4T1) breast carcinoma, and mouse (RENCA) kidney carcinoma. While Su and Ax are not currently approved for the treatment of breast carcinoma, such models have been used extensively to evaluate Su/Ax treatment impact on metastasis and resistance, by us and others [28, 30, 31, 33, 34]. Separately, mouse (3T3) fibroblast SuR cells were generated *in vitro* to compare tumor/non-tumor cell populations (see Methods for details). Using SuR/AxR cells, we performed whole transcriptome analysis to compare gene expression changes of tyrosine kinases [40] and targets of sitravatinib (identified by Patwardhan *et al*. [17]) in resistant and vehicle-treated parental (P) cells (Fig 1B). We found multiple tyrosine kinase and sitravatinib targets upregulated in resistant cells with high variability amongst cell lines observed (Fig 1B). Western blot analysis of total and phosphorylated protein levels confirmed consistent activation of key sitravatinib targets such as MET, EphA2/A3, or AXL in 4T1, RENCA, 3T3, and LM2-4 SuR or AxR cells (Fig 1C and 1D for densitometry). Interestingly, MET and AXL upregulation/activation was most common, while Eph upregulation/activation was cell-line dependent. Together, these results show consistent increases in sitravatinib targets in TKI-resistant metastatic and non-tumor cells of mouse and human origin, suggesting that sitravatinib may have activity against a broad range of complex resistant cell phenotypes.

### *In vitro* sitravatinib treatment effects are enhanced in TKI-resistant tumor and non-tumor cells

We next evaluated sitravatinib treatment impact on phosphorylation levels of MET, Eph, and AXL receptors in SuR/AxR and P cells. Mouse 4T1^AxR^, RENCA^SuR^, 3T3^SuR^, and LM2-4^SuR^ cells, typically maintained *in vitro* with Su (5μM) or Ax (0.5μM), were serum-starved for 3 hours and then treated with 4μM sitravatinib for 3 or 24 hour periods. Our results show that sitravatinib treatment decreased MET, EphA2, EphA3, or AXL protein phosphorylation levels in SuR/AxR cells after 3 hours compared to vehicle-treated control cells, indicating an enhanced effect in resistant cell lines (Fig 1C and 1E for densitometry). An exception to this was LM2-4^SuR^ cells where enhanced sitravatinib inhibitory effects in resistant cells were observed after 24 hours only, suggesting that sitravatinib sensitivity can depend on the duration of drug exposure (Fig 1C; S1 Fig for 24hr densitometry). Importantly, these results show that sitravatinib treatment can inhibit molecular targets specific to TKI resistance in P cells but have broadly enhanced effects in resistant cell populations. Together, these findings show that sensitization of cells to sitravatinib may be target-, cell-line, and time-dependent, but are consistent amongst cells with diverse resistant-mediated molecular changes.

### Proliferation inhibition by sitravatinib is increased in TKI-resistant tumor and non-tumor cells

**Fig 2:**
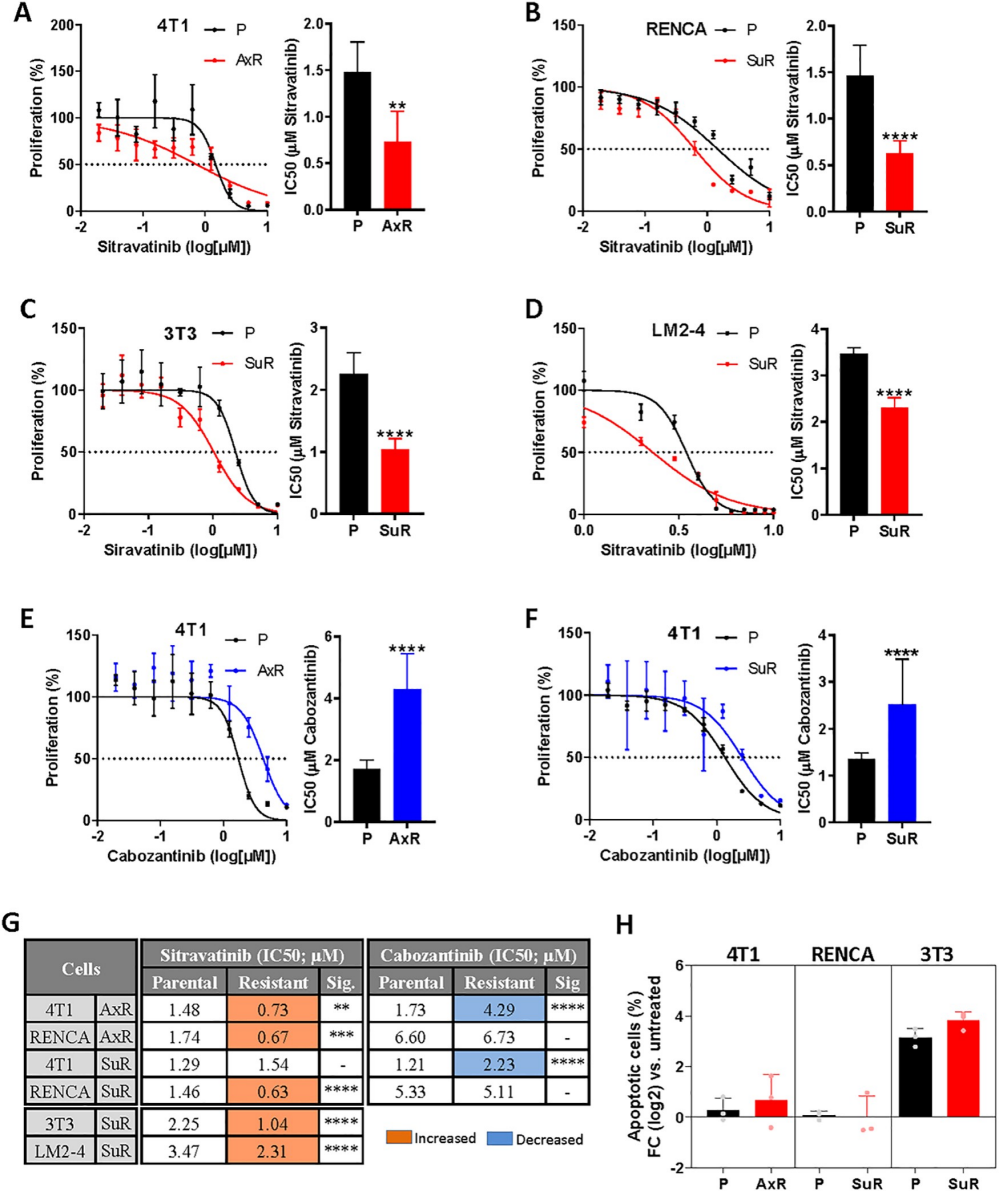
Enhanced proliferation inhibition in TKI-resistant cell lines after sitravatinib treatment. (A-F) MTS proliferation assay for (A,E) 4T1 P/AxR, (B) RENCA P/SuR, (C) 3T3 P/SuR, (D) LM2-4 P/SuR, (F) 4T1 P/SuR (n = 3–4) after 72 hours treatment with different concentrations of sitravatinib (A-D) or cabozantinib (E,F). Proliferation curves (left panel) were used to calculate IC50 values (right panel). Proliferation values were normalized to DMSO-treated vehicle controls. (G) Corresponding IC50 values from MTS proliferation assays for P or SuR/AxR cell variants following sitravatinib or cabozantinib treatment (see S2 Fig for additional MTS proliferation assays). Orange boxes indicate significantly increased sensitivity to sitravatinib and blue boxes indicate significantly decreased sensitivity to cabozantinib treatment in SuR/AxR cells compared to P controls. (H) Annexin V staining of 4T1, RENCA, and 3T3 P or SuR/AxR cell variants after 2μM sitravatinib treatment for 48 hours (n = 3) using flow cytometric analysis. Results are normalized to vehicle-treated cells. *P*, *parental; SuR*, *sunitinib-resistant; AxR*, *axitinib-resistant; FC*, *fold-change; Sig*, significance; Mean ± standard deviation (SD); ** p<0.*01*, *** *p<0*.*001*, **** *p<0*.*0001 compared to parental cells*.

Next we performed *in vitro* studies to examine sitravatinib treatment effects on cell death and division in tumor and non-tumor SuR/AxR and P cell lines. Following 72 hours of varying sitravatinib treatment concentrations, cellular proliferation was evaluated by MTS assay. We found that that the sitravatinib drug concentrations needed to reach 50% inhibition (IC50) was consistently less in SuR/AxR cells compared to P cell lines, with statistical significance reached in 5 of 6 cell lines including 4T1^AxR^, RENCA^SuR^, 3T3^SuR^, LM2-4^SuR^, and RENCA^AxR^ (Fig 2A–2D and S2A Fig, respectively; S2B Fig shows 4T1^SuR^ as an exception). In parallel, we performed comparative studies with cabozantinib, a TKI with a similar target profile. Our results show that cabozantinib IC50 concentrations in resistant and parental cells to be similar in RENCA SuR/AxR cells (S2C and S2D Fig, respectively) or increased as in 4T1 AxR/SuR cells (Fig 2E and 2F, respectively; see Fig 2G for summary). These results suggest that the enhanced sensitivity of sitravatinib in resistant cell populations may be unique compared to similar-classed drugs. In our next studies, we examined apoptosis in 4T1^AxR^, RENCA^SuR^, 3T3^SuR^ cells following 2μM sitravatinib treatment for 48 hours. Using flow cytometric analysis, we found no significant differences in apoptosis in SuR/AxR compared to P cells, suggesting that enhanced tumor-inhibitory effects of sitravatinib in TKI-resistant cells are not the result of direct cytotoxicity over short periods of drug exposure (Fig 2H). Together, these results indicate that tumor and non-tumor cell populations resistant to TKIs may be directly growth inhibited with increased potency by sitravatinib compared to treatment-naïve cell lines, but our evidence suggests this is largely caused by cytostatic, not cytotoxic, effects of the drug.

### Primary tumor inhibition effects following sitravatinib treatment are enhanced in TKI-resistant models

**Fig 3:**
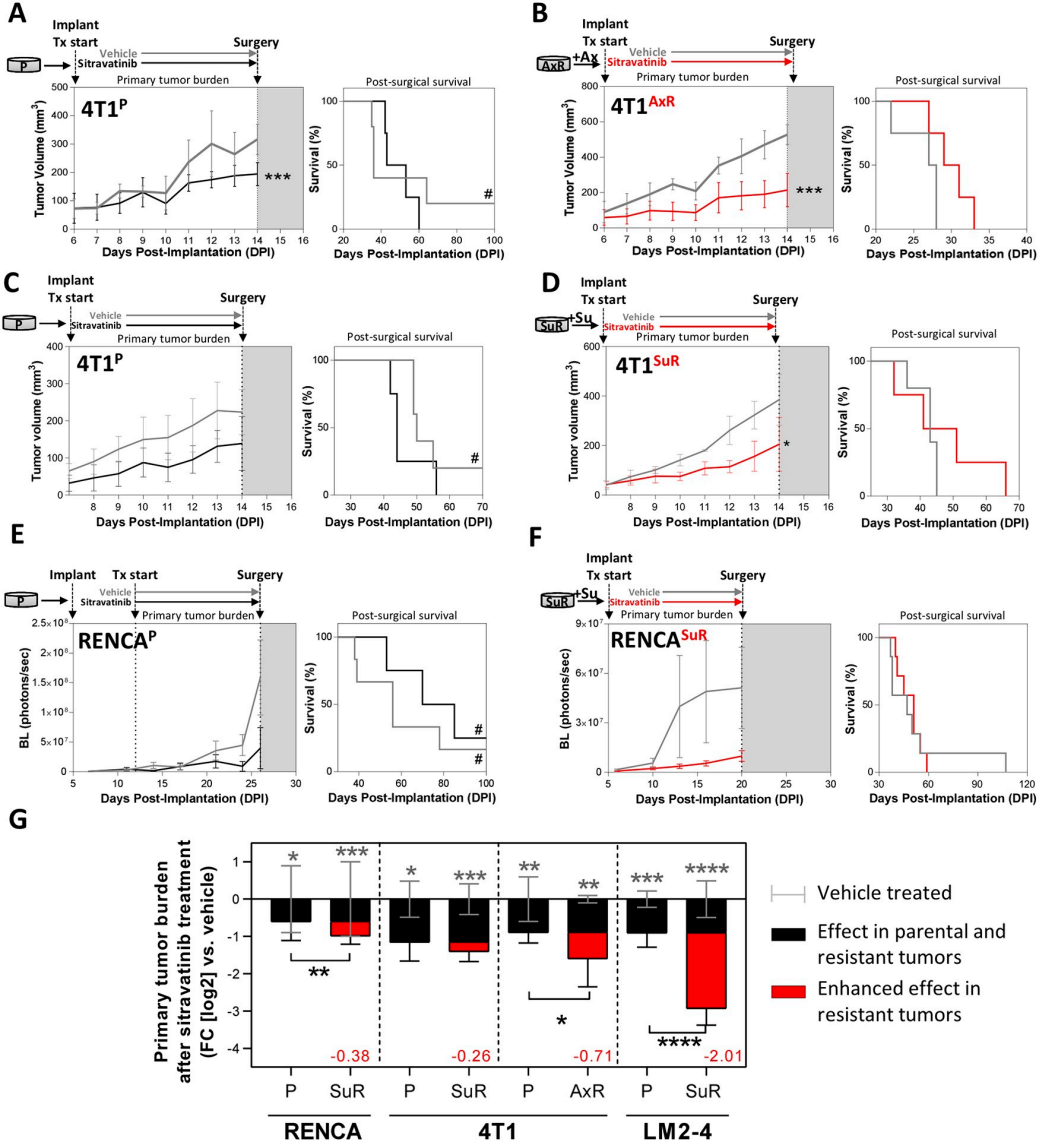
Primary tumor inhibition following sitravatinib treatment is enhanced in TKI-resistant tumor models. (A-F) Mice received orthotopic (mammary fat pad or kidney) implantation of P or SuR/AxR tumor cells. Tumor models used included 4T1^P^ and 4T1^AxR^ (Balb/c mice; n = 4–8); 4T1^P^ and 4T1^SuR^ (Balb/c mice; n = 4–5); RENCA^P^ and RENCA^SuR^ (Balb/c mice; n = 4–17). Sitravatinib was administered to mice bearing (A-B) 4T1^P^ and 4T1^AxR^, (C-D) 4T1^P^ and 4T1^SuR^, and (E-F) RENCA^P^ and RENCA^SuR^. Treatment effects were assessed for impact on primary tumor growth by tumor volume or BL measurements (left panel) and for impact on metastasis after surgical tumor removal by survival (right panel). Treatment stopped the day before surgery. (G) Analysis of orthotopic primary tumor weights (or tumor bearing kidney weight) after sitravatinib treatment (includes LM2-4 tumor model data from S3 Fig). Bar graphs represent tumor burden of sitravatinib-treated P or SuR/AxR tumor cell variants compared to treatment-naïve P control cells (*grey lines)*. Red area on bar graphs represent instances where sitravatinib treatment effect on tumor volume is enhanced in SuR/AxR tumors cell variants compared to sitravatinib-treated P tumors. Red numbers represent the enhanced effect (FC; log_2_) of sitravatinib treatment in resistant cells compared to their corresponding P cells. *Tx*, *treatment; P*, *parental; SuR*, *sunitinib-resistant; AxR*, *axitinib-resistant; Su*, *sunitinib; Ax*, *axitinib; BL*, *bioluminescence; TKI*, tyrosine kinase inhibitor; Mean ± standard deviation (SD); * p<0.*05*, ** *p<0*.*01*, *** *p<0*.*001*, **** *p<0*.*0001 compared to vehicle-treated mice*. *Significance stars in 3G*: *grey*, *represents Sitravatinib-treated vs*. *vehicle-treated; black*, *represents sitravatinib-treated SuR/AxR tumors vs*. *sitravatinib-treated P tumors*. *# represents disease-free survival until experiment end*.

We next examined sitravatinib treatment effects on localized primary tumor growth in SuR/AxR tumor models *in vivo*. To do this, we first orthotopically implanted 4T1, RENCA, or LM2-4 parental or SuR/AxR cell variants into the mammary fat pad or kidney of SCID or Balb/c mice. Following implantation, mice were then treated with sitravatinib (20 mg/kg/day) until 1 day prior to surgical resection of the primary tumor or tumor-bearing kidney, depending on the model. Our results show that sitravatinib delayed primary tumor volume as measured by calipers (breast model) or bioluminescence (BL; kidney model) in 4T1^P^/4T1^AxR^ (Fig 3A and 3B), 4T1^P^/4T1^SuR^ (Fig 3C and 3D), RENCA^P^/RENCA^SuR^ (Fig 3E and 3F), and LM2-4^P^/LM2-4^SuR^ (S3A and S3B Fig). Comparisons of resected primary tumor or kidney weights showed that sitravatinib treatment significantly inhibited tumor growth in *all* parental and TKI-resistant models compared to vehicle-treated control groups (Fig 3G). Importantly, the inhibitory effects of sitravatinib were significantly enhanced in 3 out of 4 tumor models when comparing parental and TKI-resistant models (Fig 3G; red bars). Together, these results show that sitravatinib treatment has potent inhibitory effects on localized primary tumors in multiple xenograft and isograft models, which are enhanced following antiangiogenic TKI resistance.

### Perioperative sitravatinib treatment improves survival in TKI-resistant models of spontaneous metastatic disease

**Fig 4:**
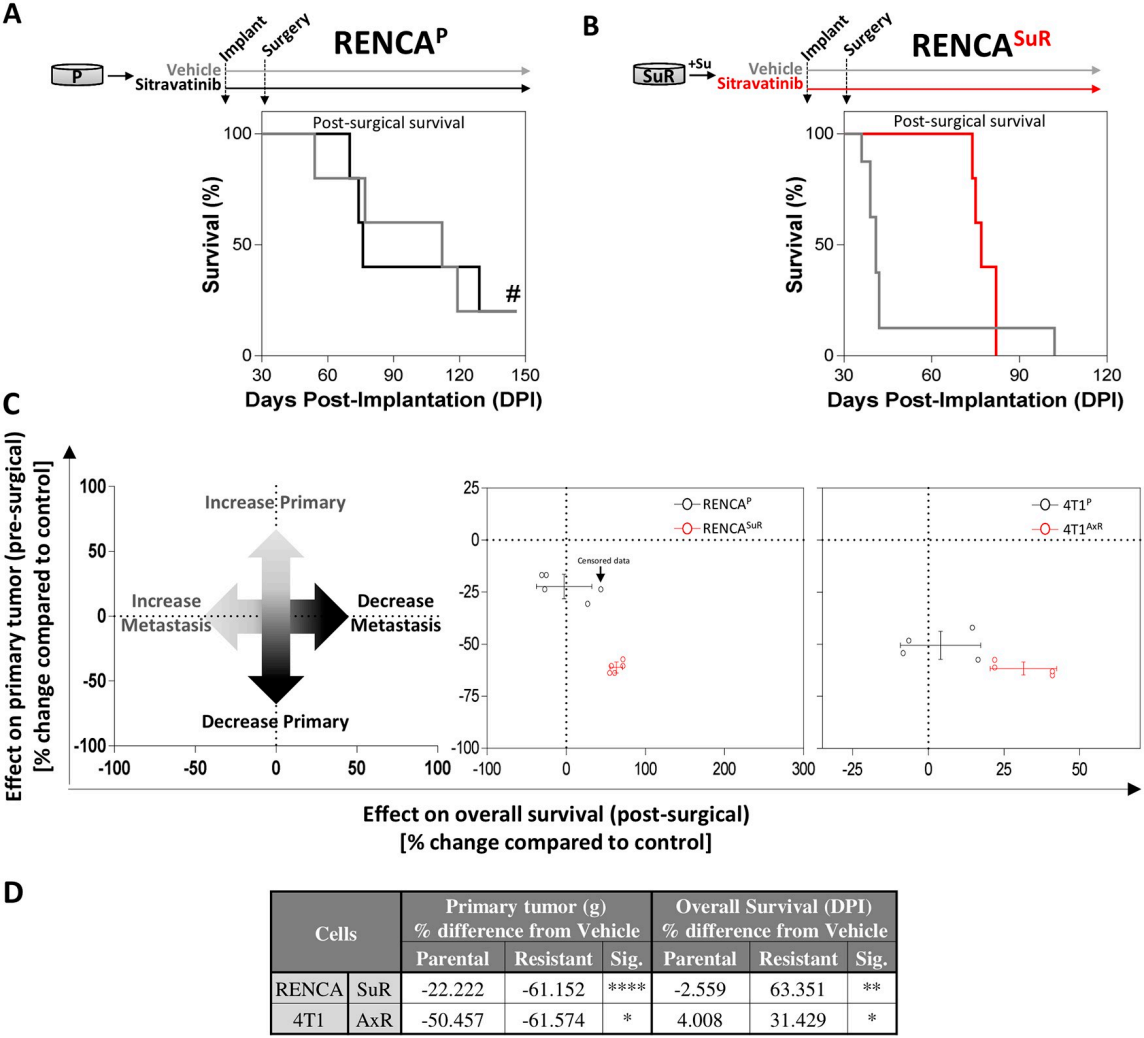
Perioperative sitravatinib treatment improves survival in TKI-resistant models of spontaneous metastatic disease. (A-B) Mice received orthotopic implantation in kidney of P or SuR tumor cells for RENCA^P^ and RENCA^SuR^ (Balb/c mice; n = 5–8) models. Sitravatinib was administered to mice bearing (A) RENCA^P^, and (B) RENCA^SuR^ tumors and stopped at the end of the experiment. Post-surgical survival is shown. (C) Combined analysis showing differences in pre-surgical primary tumor and post-surgical metastatic burden for 4T1^P^ and RENCA^P^ (black dots); and 4T1^AxR^ and RENCA^SuR^ (red dots) treated with vehicle or sitravatinib (Balb/c mice; n = 4). Crossed lines represent the standard deviation of sitravatinib-treated P (black cross) and SuR/AxR (red cross) cell-implanted tumor model data derived from comparisons of primary tumor burden data (vertical line) and median survival data (horizontal line). (D) Corresponding values for percent differences in primary tumor burden (tumor or tumor-bearing kidney weight) and overall survival (days post-implantation) are shown. Significance was assessed by student’s t test comparing SuR/AxR vs. P cell-implanted animals treated with sitravatinib and normalized to corresponding vehicle-treated animals. *Tx*, *treatment; DPI*, *days post implantation; P; parental; SuR*, *sunitinib-resistant; AxR*, *axitinib-resistant; Su*, *sunitinib; Ax*, *axitinib; TKI*, *tyrosine kinase inhibitor; g*, *grams; Sig*, *significance*. Mean ± standard deviation (SD); * p<0.*05*, ** *p<0*.*01*, *** *p<0*.*001*, **** *p<0*.*0001 compared to vehicle-treated mice or P cell-implanted model*. *# represents disease-free survival until experiment end (indicated as ‘censured’ in Fig 4C)*.

We have previously demonstrated that neoadjuvant TKI treatments in mice bearing orthotopic tumors can have potent inhibitory effects on primary tumors that do not always improve survival when treatment is stopped and tumors surgically removed [30]. We observed similar results with *presurgical-only* sitravatinib treatment in parental and TKI-resistant models where post-surgical survival was not consistently improved when treatment was stopped prior to surgery (Fig 3A–3F; right panels). A single exception to this was in the LM2-4^SuR^ tumor model where significant survival advantage was observed compared to parental tumors (S3A and S3B Fig; right panels). Therefore, to evaluate the impact of sitravatinib treatment on spontaneous metastasis and overall survival, we undertook studies where treatment was continued perioperatively (i.e., pre- and post-surgically). Importantly, we found that continuous perioperative sitravatinib treatment improved survival in RENCA or 4T1 AxR/SuR variant tumor models compared to parental controls (Fig 4A and 4B; only RENCA shown). Using pre- and post-surgical datasets from these models, we performed a combined analysis to differentiate ‘anti-primary’ and ‘anti-metastatic’ effects of sitravatinib treatment using methodology we previously optimized [30] (Fig 4C; see left panel for schematic). First, weights of surgically excised primary tumor (or tumor-bearing kidney) from sitravatinib-treated animals were normalized to their corresponding vehicle-treated groups, then these values were compared to overall survival after surgery, also normalized to their corresponding vehicle-treated groups. We found that continuous perioperative sitravatinib treatment enhanced anti-primary and anti-metastatic effects in both 4T1^AxR^ and RENCA^SuR^ cell lines (Fig 4C; middle and right panel, respectively), with comparative values shown in (Fig 4D). Importantly, in all models receiving continuous post-operative sitravatinib treatment no overt toxicity-related weight loss was observed, despite protracted periods of 120+ days (S4A–S4D Fig). Together, these results indicate that the inhibitory effects of sitravatinib treatment on primary and metastatic disease may be enhanced after prior antiangiogenic TKI treatment.

## Discussion

Patients with metastatic disease receiving antiangiogenic TKIs can have high response rates (up to 60% in mRCC) but often have short-lived efficacy, with eventual relapse common. Resistance to antiangiogenic TKIs appear to be mostly drug-specific, rather than drug-*class* specific, as TKIs with similar target profiles can show benefits in the second-line setting [6]. Such ‘cross-over’ TKI efficacy may be due to emergence of complex resistant cell phenotypes that include diverse angiogenesis and metastasis-promoting proteins such as MET and AXL [8, 13]. This raises the potential that TKIs with diverse secondary target profiles may yield benefits amongst broader resistant patient populations. Here, we examined the efficacy of sitravatinib, a novel broad-spectrum receptor TKI in resistance models that include multiple tumor and non-tumor cells of mouse and human origin, and multiple drugs (sunitinib or axitinib). Sitravatinib targets were found to be upregulated in tumor/non-tumor cell resistant cells, and sitravatinib treatment was found to enhance inhibition of cell proliferation (*in vitro*) and tumor growth (*in vivo*) following treatment failure. These findings suggest the potency of sitravatinib against diverse targets may improve overall efficacy and provide a rationale for use of sitravatinib in patients previously treated with antiangiogenic TKIs.

A key component of our studies is the use of angiogenesis inhibitor-resistant tumor cell models that consider the impact on metastatic progression. In a recent literature survey of more than 109 preclinical studies involving resistance to VEGF pathway inhibitors, we found that only a small number (<4%) examined tumor cell populations from clinically relevant metastasis that occurs after surgery [27]. In the present study, we used human and mouse cells from metastatic lesions in mice after primary tumor removal following continued treatment with axitinib or sunitinib [28]. Sitravatinib treatment had potent anti-primary (prior to surgery) and anti-metastatic (after surgery) effects depending on the duration of treatment. Our results show that these outcomes were further improved in SuR and AxR models, suggesting that prior antiangiogenic treatment does not limit sitravatinib activity in the second-line. Importantly, an early clinical evaluation of sitravatinib seems to support these conclusions. In an ongoing multi-center, open label phase 1/1b trial involving 109 patients with advanced solid tumor malignancies treated with sitravatinib, 20 ccRCC patients were identified that received VEGFR TKIs either once (48%), twice (24%), three-times (17%), or four-times (3%) [41]. Remarkably, comparisons of maximum tumor burden difference from baseline in this antiangiogenic-treated patient subset showed that 15 (75%) had prolonged stable disease and 4 (20%) had partial responses [41]. These results indicate that broad (and likely complex) resistance mechanisms resulting from TKI treatment may, at least in part, be targeted by sitravatinib. While further studies are needed to determine whether enhanced efficacy may differ amongst drug types (i.e., sunitinib vs pazopanib, etc.), or following multiple lines of prior drug exposure, these results serve as an important confirmation that broad-target TKIs may improve the treatment of complex resistant phenotypes that emerge after VEGF pathway blockade.

In this regard, complexity of reported mechanisms that explain antiangiogenic drug resistance have rapidly increased to include several compensatory pathways (i.e., FGF, PDGF, and among others), recruitment of bone-marrow derived cell populations, metabolomics, and, more recently, activation/suppression of immune-regulatory components (summarized in [42]). We and others have shown that, in some instances, these resistant mechanisms can include promotion of metastatic phenotypes [43]. This may be driven, at least in part, by increases in MET signaling, but other mechanisms such as increased Eph signaling may also play a role. Indeed, upregulation of EphA2 or ephrin-A1 may be involved in resistance to VEGF-targeted therapies [44], and recent evidence suggests that EphB4 overexpression can confer resistance to sunitinib treatment [45]. Our present studies demonstrate EphA2 or EphA3 can be upregulated or activated following resistance, but this effect can be variable amongst several cell lines. This may explain why broad Eph-signaling blockade by broad-spectrum inhibitors such as sitravatinib may improve outcomes compared to more specific approaches; however, further testing is required to evaluate this question. Related to this, another key consideration is whether resistance derives from tumor or host stromal cells, or both together in concert. There is increasing evidence, by us and others, demonstrating that the anti-tumor effects of VEGFR TKIs—thought to predominantly be due to antiangiogenic properties—also have direct effects on the tumor [28, 46]. In our present studies, we demonstrate key molecular and proteomic changes in resistant tumor and non-tumor cell populations that include increases in RTK- and sitravatinib-specific targets, suggesting that sitravatinib treatment efficacy after resistance may be related to enhanced effects on tumor and host cell populations.

A key consideration of our study is that we examined treatments as a monotherapy following TKI resistance, yet it may be the case that the most effective clinical use of sitravatinib is in combination with other drugs. For instance, sitravatinib is currently being evaluated clinically in oral cavity cancer, NSCLC, and mRCC patients in combination with nivolumab, an immune checkpoint inhibitor targeting the PD-1 pathway. Thus far, the potential benefits of TKI/PD-1 drug combinations have been mixed, in large part because of variable toxicities. For example, in mRCC drug combinations of axitinib with pembrolizumab or avelumab (PD-1 and PD-L1 antibodies, respectively) have demonstrated acceptable toxicity and anti-tumor activity [47, 48], while the combination of pazopanib and pembrolizumab was not tolerated leading to trial termination [49]. This variability of side-effects may stem from drug selectivity and “off-target” effects, along with differing target profiles [8]. Currently, it is unknown whether sitravatinib/PD-1 pathway inhibitor combinations will be efficacious, but it is notable that early indications suggest favorable toxicities profiles in most patients [19, 50].

It is also possible that sitravatinib may have direct action on immune-regulatory cells that may explain efficacy in TKI-refractory settings. Uniquely, sitravatinib is active in inhibiting TAM family receptors, which are activated by their cognate ligands Gas6 and PROS1 [20]. TAM receptors/ligands are overexpressed in numerous tumor types, can correlate with tumor stage and chemo/radio-resistance, and serve as inhibitory receptors on infiltrating immune cells in the tumor microenvironment that suppress host antitumor immunity [51, 52]. Importantly, TAM receptors can regulate adaptive immune responses, inflammation, and tissue-repair promotion mechanisms—all modulators of metastatic behavior [52–54]. While our studies provide confirmation that AXL is upregulated following TKI resistance; further exploration is required to determine if the tumor and metastasis-limiting effects of sitravatinib are the result of TAM family inhibition.

Overall our studies demonstrate a therapeutic advantage for sitravatinib in TKI resistant tumor cells *in vitro* and *in vivo*. While efficacious in a non-resistant setting, sitravatinib can prolong survival in resistant tumors. Together, our findings supporting further clinical investigation for the use of sitravatinib in patients that have failed on antiangiogenic therapy.

## Supporting information

S1 Appendix Uncropped original western blots. (PDF)

S2 Appendix Data used to generate figures. (XLSX)

S1 Fig Tyrosine kinase phosphorylation after 24 hours of sitravatinib treatment. Densitometric analysis of western blots for relative phosphorylation levels shown in Fig 1C comparing P and SuR/AxR cell variants after treatment with sitravatinib at 4μM for 24 hours in 4T1, RENCA, 3T3 and LM2-4 SuR or AxR cell variants. Protein levels were normalized to α-tubulin and compared to vehicle (DMSO) treated cells. Red area in bar graphs represent instances where sitravatinib treatment effect on phosphorylation levels is enhanced in SuR or AxR cells compared to sitravatinib treatment effect in P cells. Red numbers represent the enhanced effect (FC; log2) in SuR or AxR cells compared to P cells after sitravatinib treatment. *P*, *parental; SuR*, *sunitinib resistant; AxR*, *axitinib resistant; FC*, *fold-change; n = 1*, *No error bars shown*, *n = 2*, *gray error bars shown; n>2*, *black error bars shown*. *Mean ± standard deviation (SD)*.
(TIFF)

S2 Fig Effects of sitravatinib and cabozantinib treatment on proliferation in TKI-resistant cell lines. (A-D) MTS proliferation assay for (A) RENCA P/AxR, (B) 4T1 P/SuR, (C) RENCA P/SuR, and (D) RENCA P/AxR (n = 3–4) after 72 hours of varying sitravatinib (A,B) or cabozantinib (C,D) concentrations. Proliferation curves (left panel) were used to calculate IC50 (right panel). Proliferation values were normalized and compared to vehicle (DMSO)-treated control cells. *P*, *parental; SuR*, *sunitinib-resistant; AxR*, *axitinib-resistant; TKI*, *tyrosine kinase inhibitors*. Mean ± standard deviation (SD); *** p<0.*001*, *compared to parental cells*.
(TIFF)

S3 Fig Primary tumor inhibition following sitravatinib treatment is enhanced in a TKI-resistant metastatic LM2-4 tumor model. (A-B) Sitravatinib was administered to SCID mice (n = 4–18) bearing (A) LM2-4^P^ and (B) LM2-4^SuR^ tumors following orthotopic implantation. Treatment effects were assessed for impact on primary tumor growth by tumor volume measurements (left panel) and for impact on metastasis after surgical tumor removal by survival (right panel). Treatment stopped the day before surgery. *Tx*, *treatment; P*, *parental; SuR*, *sunitinib-resistant; Su*, sunitinib; Mean ± standard deviation (SD); * p<0.*05*, *** *p<0*.*001*, **** *p<0*.*0001 compared to vehicle-treated mice*. *# represents disease-free survival until experiment end*.
(TIFF)

S4 Fig Mouse body weights after sitravatinib treatment. (A-D) Body weights from experiments presented in Fig 4 that included mice receiving continuous sitravatinib treatment after the surgical removal of RENCA and 4T1 P or SuR/AxR tumor variants until endpoint or experiment termination. Tumor models (A) RENCA^P^, (B) RENCA^SuR^, (C) 4T1^P^, and (D) 4T1^AxR^. *P*, *parental; SuR*, *sunitinib-resistant; AxR*, *axitinib-resistant; Su*, *sunitinib; Ax*, *axitinib; g*, *grams; Tx*, Treatment; *, *represents end-stage disease criteria endpoint reached; #*, *represents disease-free survival until experiment end*.
(TIFF)
