# Supplementary material for: Enhanced efficacy of sitravatinib in metastatic models of antiangiogenic therapy resistance
Source: PLoS One. 2019 Aug 1;14(8):e0220101. doi: 10.1371/journal.pone.0220101 (PMC6675057; doi:10.1371/journal.pone.0220101)

S1 APPENDIX:

Full uncropped western blots for Figure 1C

Full uncropped blots: Figure 1C

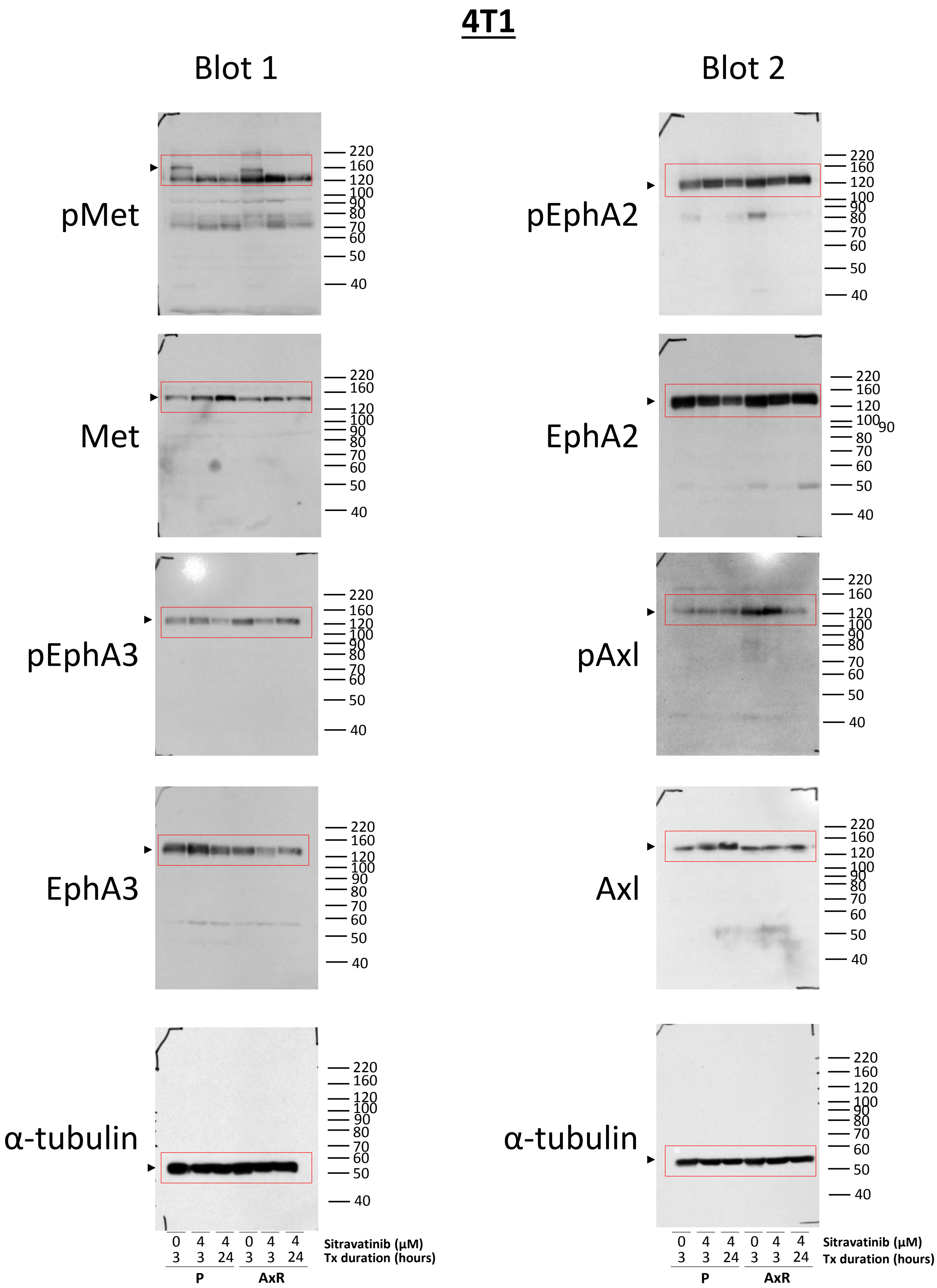

Full uncropped blots: Figure 1C

RENCA

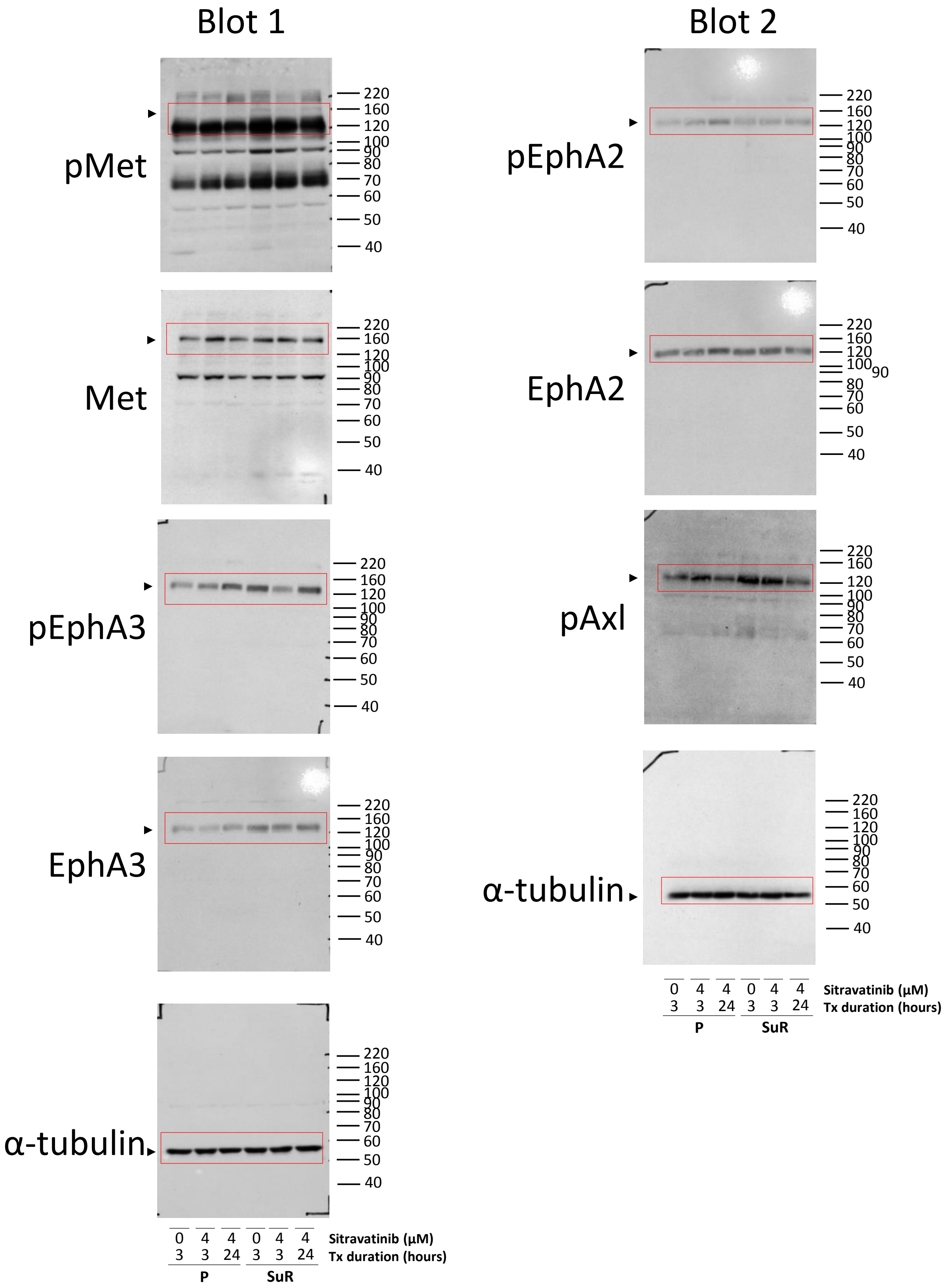

Full uncropped blots: Figure 1C

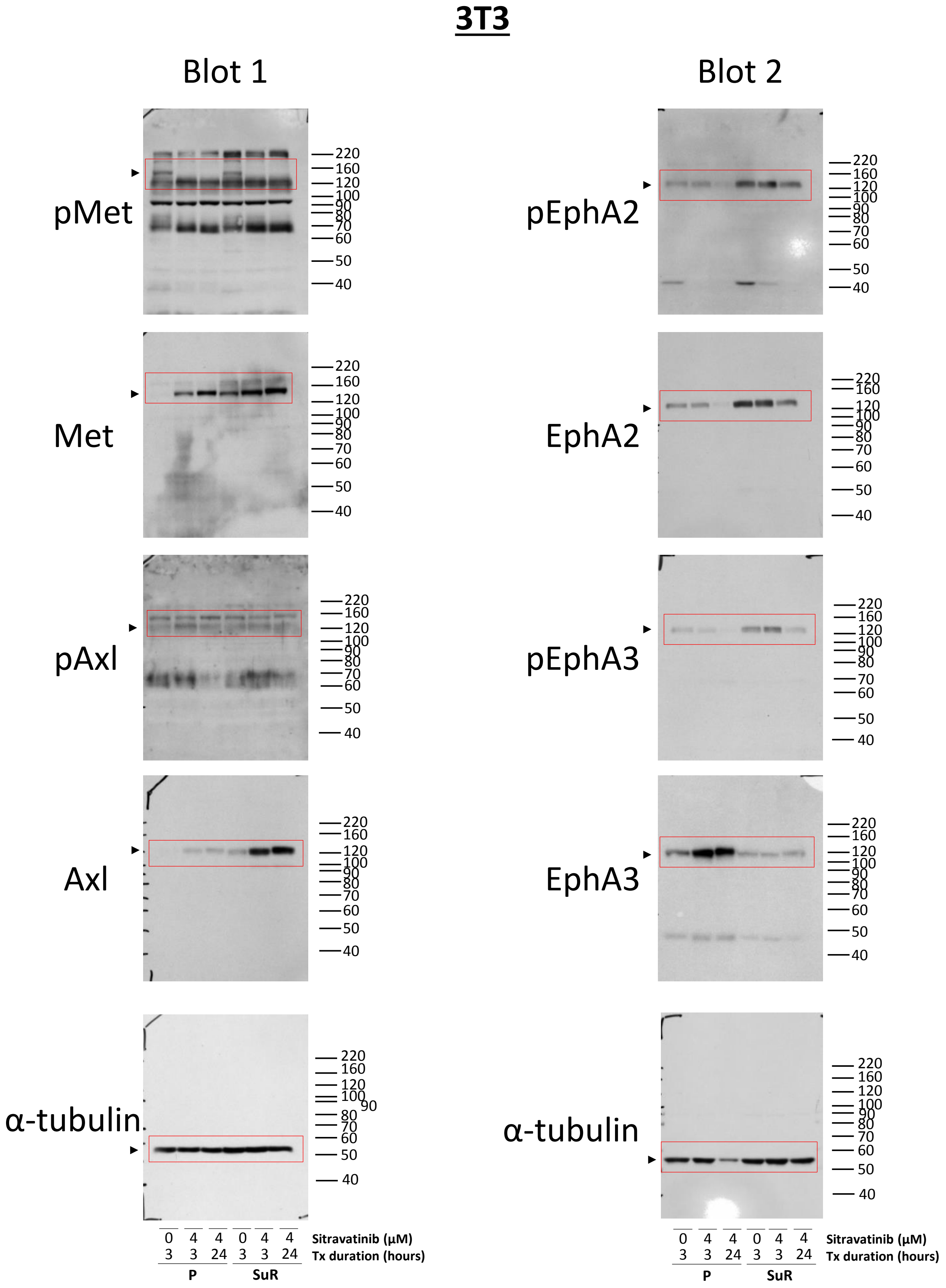

Full uncropped blots: Figure 1C

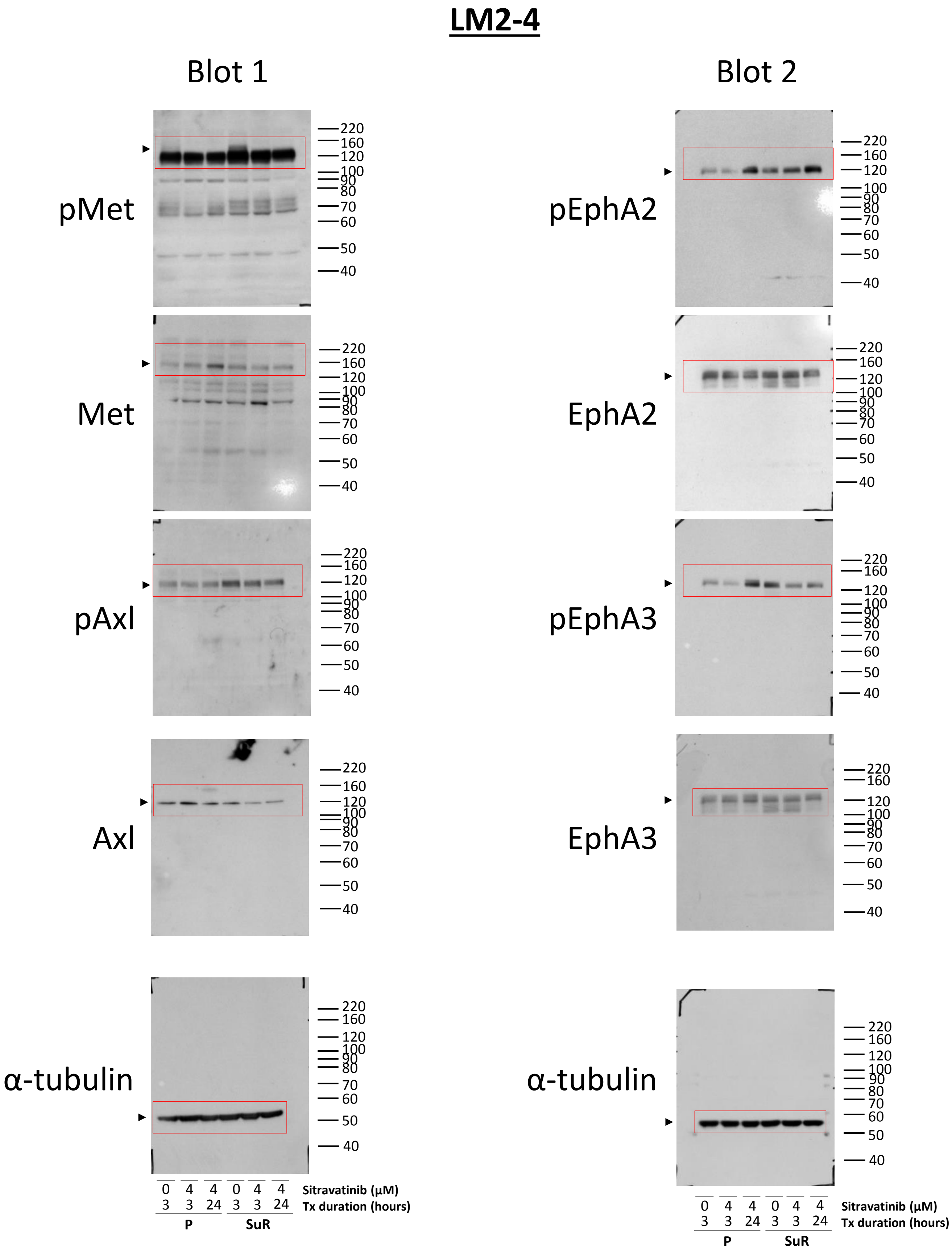

Supplement: S1 Appendix — (PDF) [file pone.0220101.s001.pdf]
