# Supplementary figures and images for: Enhanced efficacy of sitravatinib in metastatic models of antiangiogenic therapy resistance
Source: PLoS One. 2019 Aug 1;14(8):e0220101. doi: 10.1371/journal.pone.0220101 (PMC6675057; doi:10.1371/journal.pone.0220101)

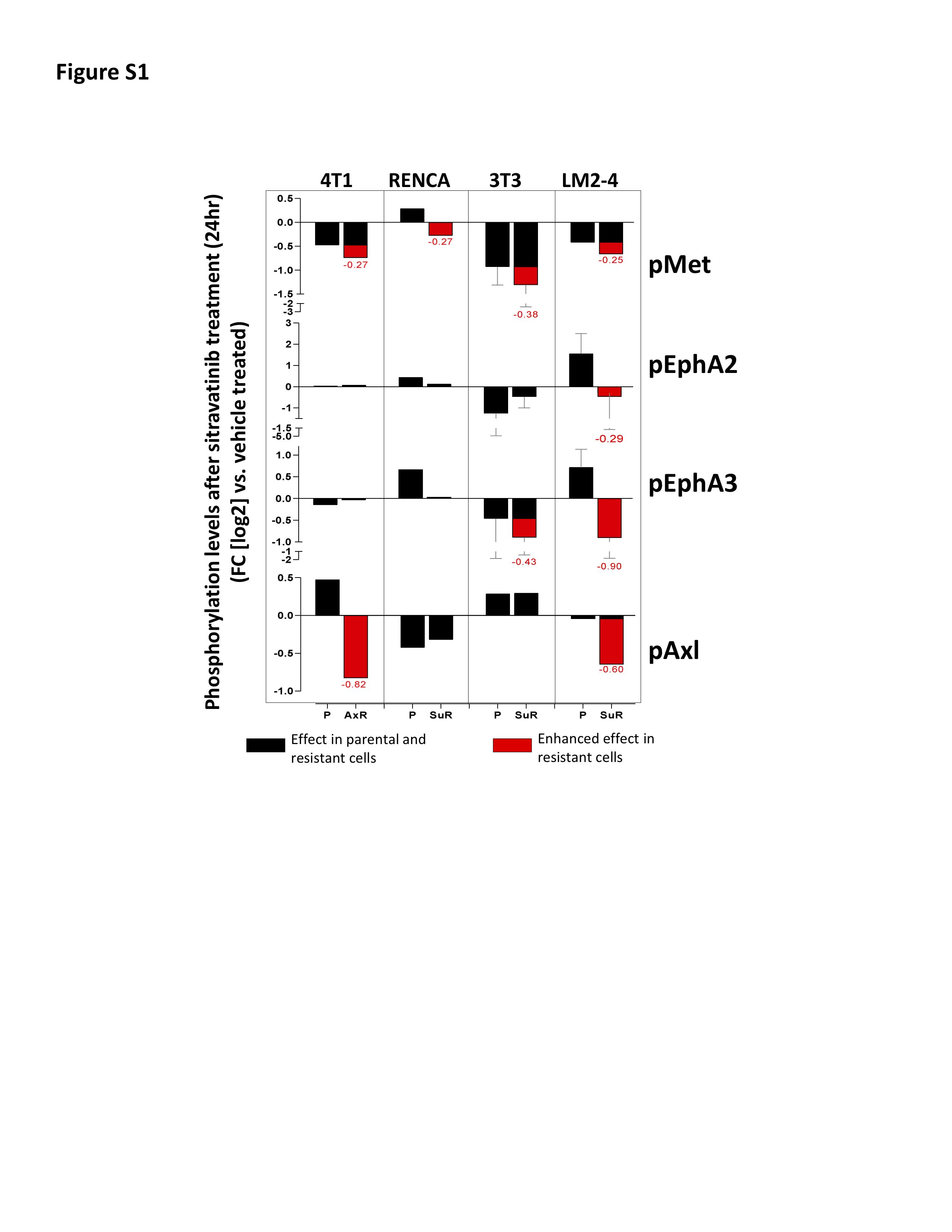

Supplement: S1 Fig — Densitometric analysis of western blots for relative phosphorylation levels shown in Fig 1C comparing P and SuR/AxR cell variants after treatment with sitravatinib at 4μM for 24 hours in 4T1, RENCA, 3T3 and LM2-4 SuR or AxR cell variants. Protein levels were normalized to α-tubulin and compared to vehicle (DMSO) treated cells. Red area in bar graphs represent instances where sitravatinib treatment effect on phosphorylation levels is enhanced in SuR or AxR cells compared to sitravatinib treatment effect in P cells. Red numbers represent the enhanced effect (FC; log2) in SuR or AxR cells compared to P cells after sitravatinib treatment. P, parental; SuR, sunitinib resistant; AxR, axitinib resistant; FC, fold-change; n = 1, No error bars shown, n = 2, gray error bars shown; n>2, black error bars shown. Mean ± standard deviation (SD). (TIFF) [file pone.0220101.s003.tiff]

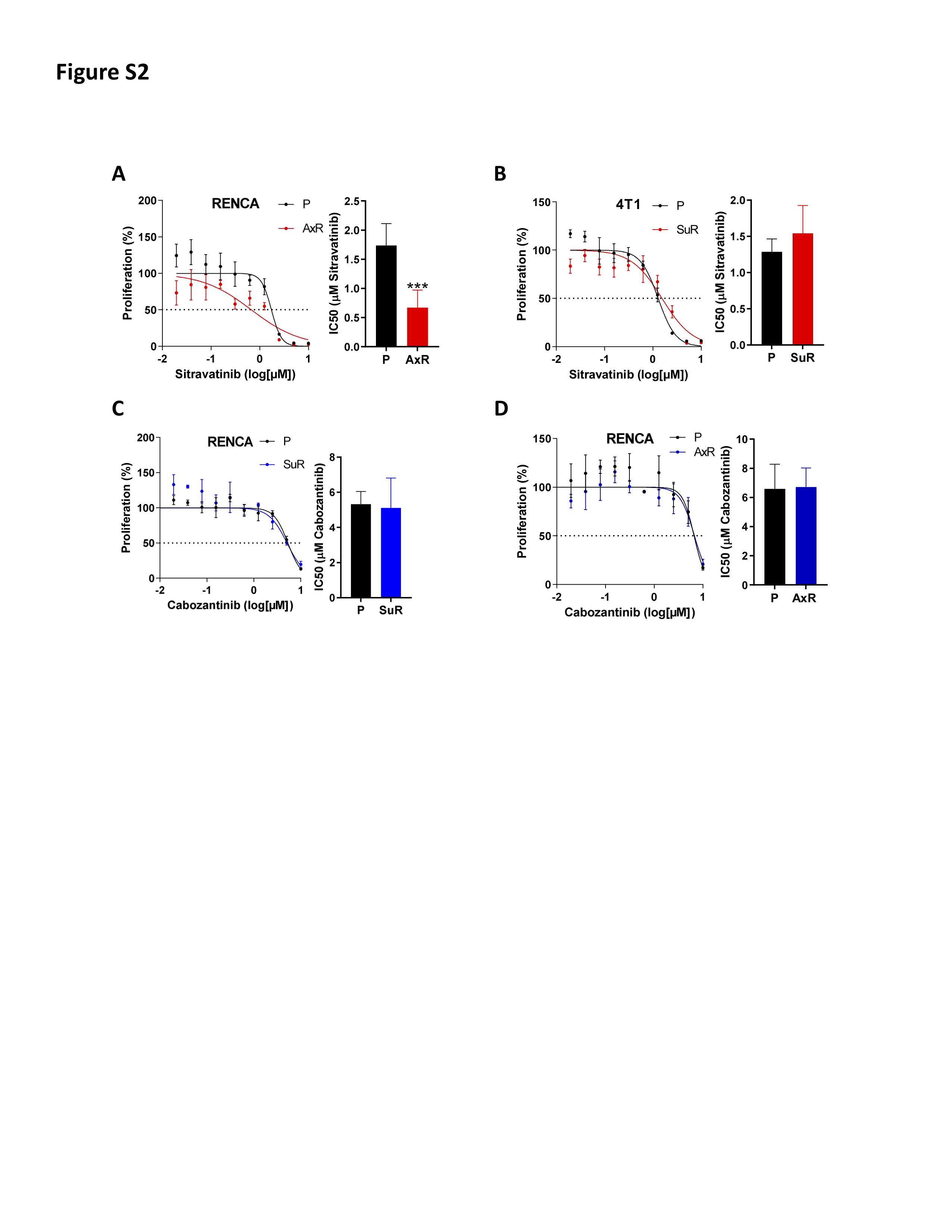

Supplement: S2 Fig — (A-D) MTS proliferation assay for (A) RENCA P/AxR, (B) 4T1 P/SuR, (C) RENCA P/SuR, and (D) RENCA P/AxR (n = 3–4) after 72 hours of varying sitravatinib (A,B) or cabozantinib (C,D) concentrations. Proliferation curves (left panel) were used to calculate IC50 (right panel). Proliferation values were normalized and compared to vehicle (DMSO)-treated control cells. P, parental; SuR, sunitinib-resistant; AxR, axitinib-resistant; TKI, tyrosine kinase inhibitors. Mean ± standard deviation (SD); *** p<0.001, compared to parental cells. (TIFF) [file pone.0220101.s004.tiff]

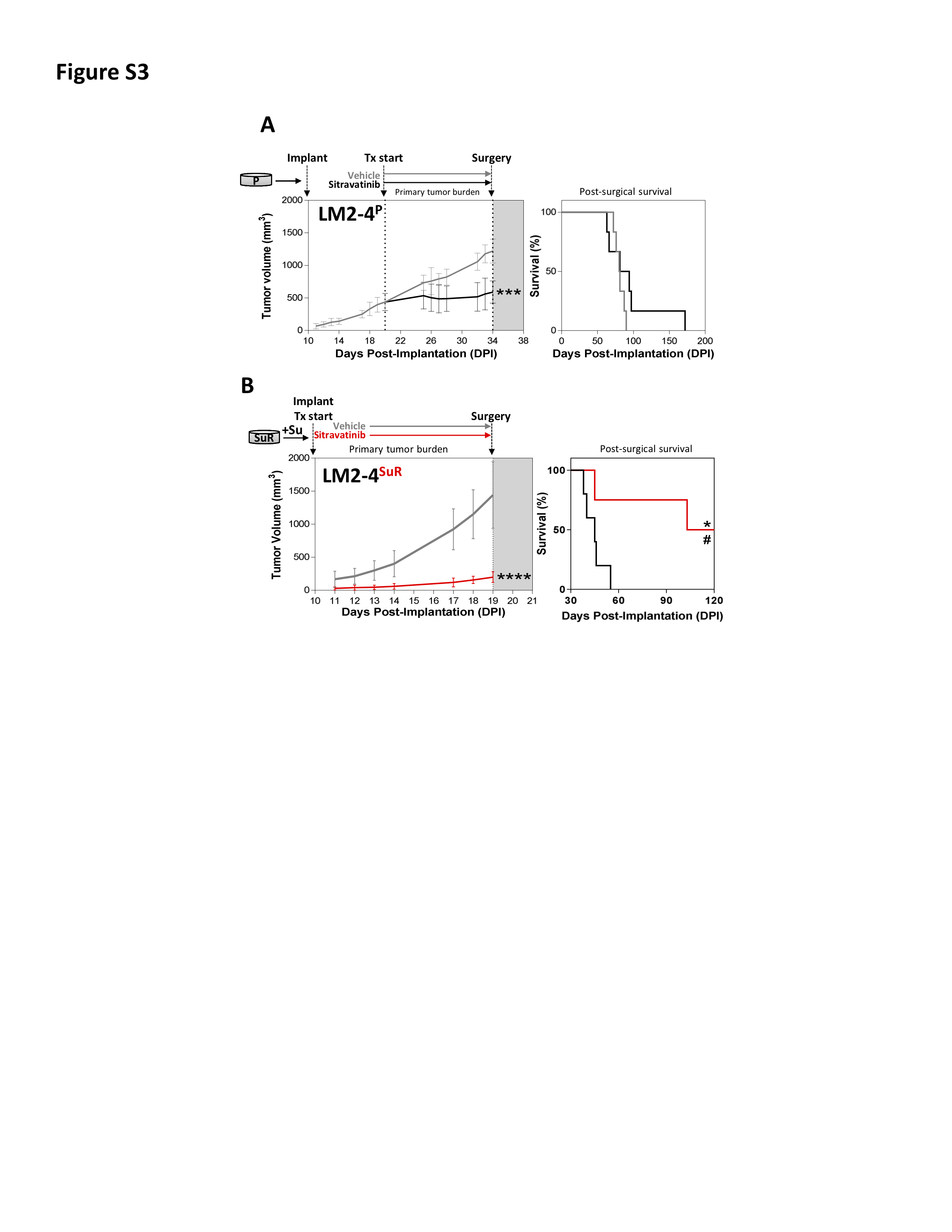

Supplement: S3 Fig — (A-B) Sitravatinib was administered to SCID mice (n = 4–18) bearing (A) LM2-4P and (B) LM2-4SuR tumors following orthotopic implantation. Treatment effects were assessed for impact on primary tumor growth by tumor volume measurements (left panel) and for impact on metastasis after surgical tumor removal by survival (right panel). Treatment stopped the day before surgery. Tx, treatment; P, parental; SuR, sunitinib-resistant; Su, sunitinib; Mean ± standard deviation (SD); * p<0.05, *** p<0.001, **** p<0.0001 compared to vehicle-treated mice. # represents disease-free survival until experiment end. (TIFF) [file pone.0220101.s005.tiff]

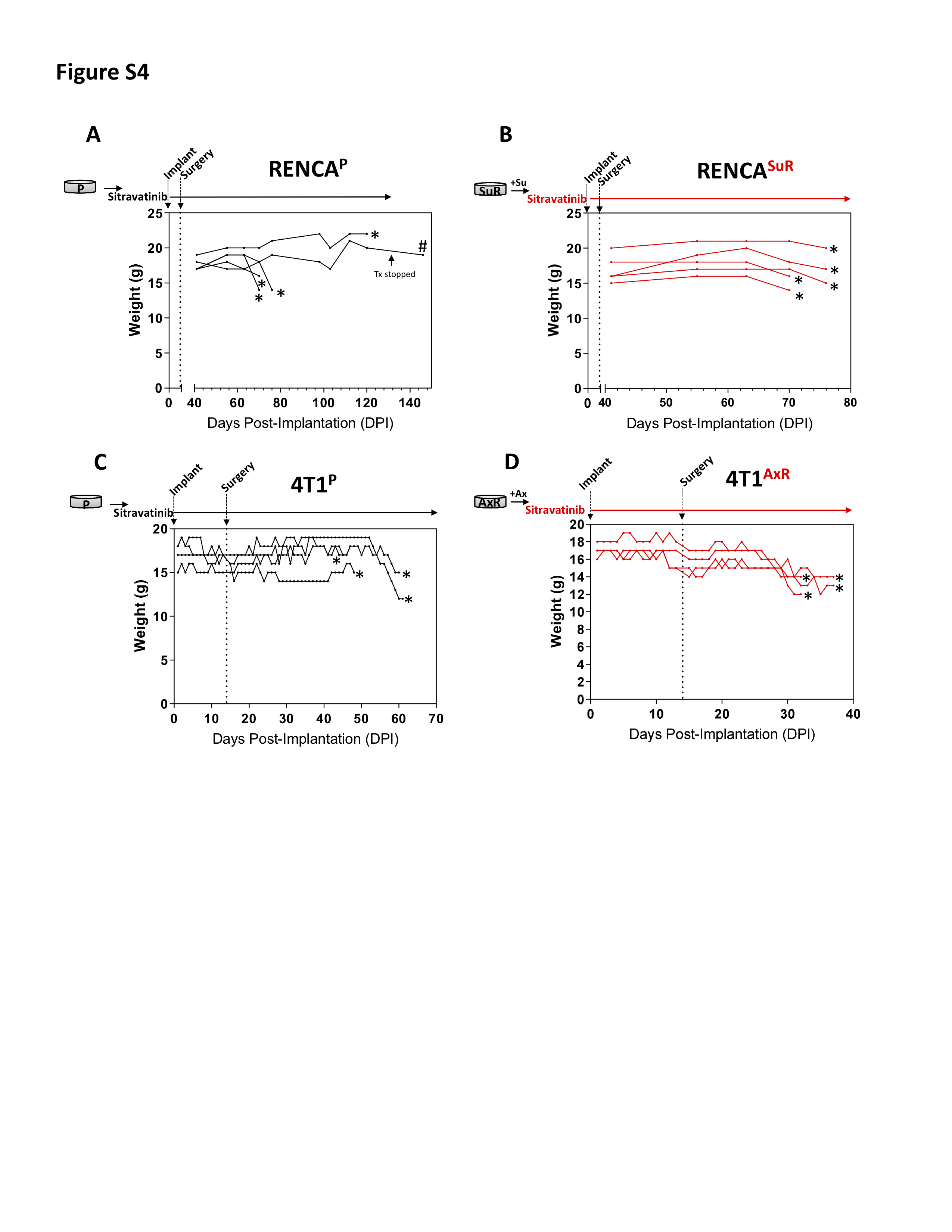

Supplement: S4 Fig — (A-D) Body weights from experiments presented in Fig 4 that included mice receiving continuous sitravatinib treatment after the surgical removal of RENCA and 4T1 P or SuR/AxR tumor variants until endpoint or experiment termination. Tumor models (A) RENCAP, (B) RENCASuR, (C) 4T1P, and (D) 4T1AxR. P, parental; SuR, sunitinib-resistant; AxR, axitinib-resistant; Su, sunitinib; Ax, axitinib; g, grams; Tx, Treatment; *, represents end-stage disease criteria endpoint reached; #, represents disease-free survival until experiment end. (TIFF) [file pone.0220101.s006.tiff]
